# Differentiated HIV services for transgender people in four South African districts: population characteristics and HIV care cascade

**DOI:** 10.1002/jia2.25987

**Published:** 2022-10-12

**Authors:** Rutendo Bothma, Cara O'Connor, Jolie Nkusi, Vusi Shiba, Jacob Segale, Luyanda Matsebula, J. Joseph Lawrence, L. Leigh‐Ann van der Merwe, Matthew Chersich, Naomi Hill

**Affiliations:** ^1^ Wits Reproductive Health and HIV Institute (Wits RHI) University of the Witwatersrand Johannesburg South Africa; ^2^ United States Agency for International Development/Southern Africa City of Tshwane South Africa; ^3^ Social, Health & Empowerment Feminist Collective of Transgender Women of Africa East London South Africa

**Keywords:** differentiated service delivery models, gender‐affirming, HIV services, hormone therapy, South Africa, transgender

## Abstract

**Introduction:**

Transgender people in South Africa are disproportionately affected by HIV, discrimination and stigma. Access to healthcare and health outcomes are poor. Although integrating gender‐affirming healthcare with differentiated HIV prevention, care and treatment services has shown improvement in HIV service uptake and health outcomes among transgender people, evidence is lacking on the implementation of differentiated service delivery models in southern Africa. This article describes a differentiated service delivery model across four South African sites and transgender individuals who access these services. We assess whether hormone therapy (HT) is associated with continued use of pre‐exposure prophylaxis (PrEP) and viral load suppression.

**Methods:**

In 2019, differentiated healthcare centres for transgender individuals opened in four South African districts, providing gender‐affirming healthcare and HIV services at a primary healthcare level. Routine programme data were collected between October 2019 and June 2021. Descriptive statistics summarized patient characteristics and engagement with HIV prevention and treatment services. We conducted a multivariate logistic regression analysis to determine whether HT was associated with viral load suppression and PrEP continued use.

**Results:**

In the review period, we reached 5636 transgender individuals through peer outreach services; 86% (4829/5636) of them accepted an HIV test and 62% (3535/5636) were linked to clinical services. Among these, 89% (3130/3535) were transgender women, 5% (192/3535) were transgender men and 6% (213/3535) were gender non‐conforming individuals. Of those who received an HIV test, 14% (687/4829) tested positive and 91% of those initiated antiretroviral treatment. Viral load suppression was 75% in this cohort. PrEP was accepted by 28% (1165/4142) of those who tested negative. Five percent (161/3535) reported ever receiving HT through the public healthcare system. Service users who received HT were three‐fold more likely to achieve viral load suppression. We did not find any association between HT and continued use of PrEP.

**Conclusions:**

A differentiated HIV and gender‐affirming service delivery model at a primary healthcare level is feasible and can enhance service access in South Africa. HT can improve HIV clinical outcomes for transgender people. As trust is established between the providers and population, uptake of HIV testing and related services may increase further.

## 1 INTRODUCTION

South Africa bears 19% of the global HIV burden, with over 7.7 million people living with HIV [1]. The risk for HIV varies considerably between population groups, and globally transgender people—those whose gender identity differs from their birth‐assigned sex [2]—are among those at highest risk [3, 4]. The increased burden of HIV is largely due to economic and social marginalization and structural factors that affect transgender people, such as poor access to services, experience of discrimination, violence and human rights violations, as well as the prevalence of risk behaviours [5].

In 2017, the South African National AIDS Council developed the South African National Lesbian, Gay, Bisexual, Transgender and Intersex (LGBTI) HIV Plan 2017–2022 in response to the global call to reach key and vulnerable populations with targeted interventions [6], recognizing transgender people as a key population in South Africa's HIV response for the first time. Modelling data suggest that for HIV incidence to fall below the AIDS elimination threshold, that is incidence of less than 0.1%, South Africa needs to markedly scale up HIV prevention among key populations, including transgender people [7].

HIV prevalence among the South African general population aged 15–49 is 19.1% [8], while prevalence among transgender women (TGW) is much higher: 63% in Johannesburg and approximately 50% in Cape Town and Buffalo City [9]. Globally HIV prevalence among transgender men is 10 times that of the general population [10, 11]; however, the prevalence in transgender men in South Africa is unknown. In South Africa, there are an estimated 87,214 TGW, 28,065 transgender men and 66,076 gender non‐conforming individuals, about 0.3% of the total population [12].

The Joint United Nations Programme on HIV/AIDS (UNAIDS) has recognized that comprehensive, people‐centred services are pivotal to ending AIDS by 2030 [13]. Reaching this global goal will require 90% of transgender people to have access to *“HIV services differentiated with or linked to sexually transmitted infections (STIs), mental health, gender‐affirming therapy, IPV [intimate partner violence] programmes, and SGBV [sexual‐ and gender‐based violence] programmes that include PEP, emergency contraception and psychological first aid”* by 2025. Similarly, the World Health Organization and the International AIDS Society have called for a differentiated service delivery model with a people‐centred approach to engage transgender people in health services and decrease treatment interruption [14, 15].

Global studies have shown that when HIV services are provided within a gender‐affirming healthcare approach, including hormone therapy (HT), pre‐exposure prophylaxis (PrEP) acceptability among TGW increases [16, 17] and continuity of treatment and viral suppression rates for transgender people on antiretroviral therapy (ART) strengthens [18, 19, 20]. In South Africa, access to gender‐affirming healthcare in the public sector is limited, with HT provision limited to a handful of tertiary hospitals.

Our study describes a gender‐affirming healthcare intervention in which HIV prevention and treatment services and HT were delivered in a differentiated HIV service delivery model at a primary healthcare level. Differentiated service delivery offers stigma‐free services adapted to the needs of transgender people who may require specialized services, such as community ART and PrEP refills, one‐stop services which address HIV and other health issues, involvement of transgender peers for service delivery and flexible clinic hours [14]. The study describes the characteristics of the TGW, transgender men and gender non‐conforming people who received services from the programme. These services were gender‐affirming, where service delivery integrates transgender individuals’ mental, physical, social and health needs, while respecting their self‐identified gender [21]. We report on the services they received through the programme and their clinical outcomes and assess whether the provision of HT was associated with continued use of PrEP or viral load suppression.

## 2 METHODS

### 2.1 Study setting and programmatic model

**Figure 1:**
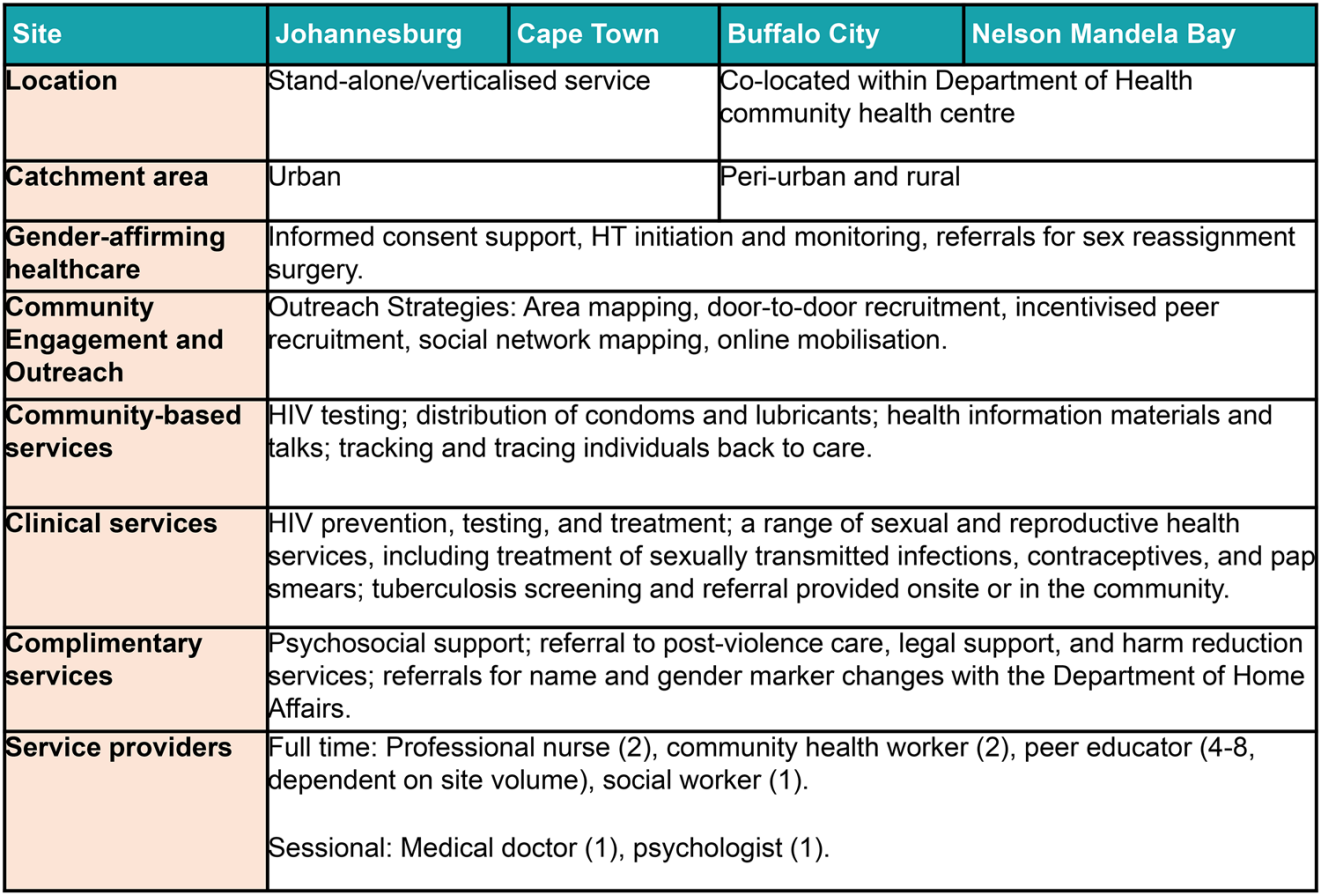
Wits RHI differentiated transgender healthcare model. Abbreviations: HT, hormone therapy; Wits RHI, the Wits Health and HIV Research Institure.

The Wits Health and HIV Research Institute (Wits RHI) is a multi‐disciplinary research institute of the University of the Witwatersrand, Johannesburg. In 2019, the Wits RHI key populations programme opened healthcare centres for transgender people in four South African districts—Cape Town, Johannesburg, Buffalo City and Nelson Mandela Bay. The first two are stand‐alone sites in large urban centres, and the last two are co‐located within public health facilities in smaller towns which serve remote rural areas (Figure 1). These centres, funded by the U.S. President's Emergency Plan for AIDS Relief (PEPFAR) through the U.S. Agency for International Development (USAID) Southern Africa, are the first U.S. Government‐funded centres addressing the specific needs of transgender people in sub‐Saharan Africa and build on global lessons in gender‐affirming healthcare [22, 23, 24].

Our differentiated service delivery model included multi‐disciplinary teams who provided gender‐affirming healthcare, including HT for adult transgender and gender non‐conforming people. Individuals younger than 18 years were referred to public and private health services. Services were provided on site or through mobile clinics equipped with gazebos, which enabled service provision on streets, in informal settlements and in the *veld* (open fields). Transgender peer outreach workers (who were trained to provide outreach services) recruited and retained service users in care. The clinical team initiated ART for HIV‐positive individuals, usually on the same day of HIV diagnosis. Viral load testing was conducted after 6 months of ART initiation, at 12 months and annually thereafter for virally suppressed clients. Clients who were not virally suppressed had viral load testing more frequently according to the South African guidelines [25]. Nurses offered PrEP to anyone who tested HIV negative, empowering service users with the choice to take PrEP based on their own assessment of their HIV risk.

Our programme is unable to fulfil the demand for HT, with over 200 transgender individuals on a waiting list to receive this service. HT was added to the South African Department of Health Essential Medicines List in December 2019, with the indication that it should be prescribed by doctors in tertiary settings. Therefore, there is a limited stock made available in district pharmacies to provide for clients referred by doctors for maintenance of care in primary healthcare facilities, as in our sites.

### 2.2 Data collection

Peer educators, social workers and clinicians collected data at service delivery points using standardized forms for outreach, behavioural risk assessment, demographic and clinical services information. Information on sexual and behavioural risk was collected during peer outreach activities. Demographic data were collected only when clients were enrolled in clinical services and included information about HIV status and past experiences utilizing gender‐affirming healthcare. Paper‐based data were captured into a Research Electronic Data Capture (REDCap) database [26, 27]. Our programme ensured that data collection and management complied with the provision of the South African Protection of Personal Information Act (4 of 2013) [28].

### 2.3 Study design and measures

This retrospective record review utilized routine programme data collected between October 2019 and June 2021 at the four transgender healthcare centres. We describe the number of community outreach contacts, sexual and behavioural risks of HIV infection and socio‐demographic characteristics. We present the HIV prevention and treatment cascade, HT initiations and examine associations between HT and HIV clinical outcomes.

### 2.4 Data analysis

Using STATA 15, we conducted a descriptive data analysis of the characteristics of the study population. All transgender people who received services at the four programme sites between the specified dates were included. Categorical variables were described with proportions and continuous variables were presented as means with standard deviations. In instances of incomplete or missing data, all available data points were included. The denominator for each proportion is the total number of respondents for whom the relevant data collection tool was completed, with non‐response included as a category for each variable. We examined whether HT was associated with key HIV clinical outcomes: continuous use of PrEP for 10 months among PrEP users and viral load suppression among clients on ART for 6 months or more. Crude odds ratios (ORs) were calculated with logistic regression. Then, factors significant at *p*‐value <0.1 on univariate analysis were included in a multivariate logistic regression analysis. Variables were excluded from the multivariate if there was collinearity or reverse causality.

### 2.5 Ethical considerations

Ethical clearance for the use of routine programmatic data for research purposes was obtained from the University of the Witwatersrand Human Research Ethics Committee (protocol number M190428). Individual consent was waivered for this record review as data were presented at an aggregate level.

## 3 RESULTS

### 3.1 Characteristics of service users

#### 3.1.1 Socio‐demographic characteristics

The programme reached 5636 transgender individuals through peer outreach. Of the 5636 individuals, 62% (3535/5636) were linked to a professional nurse for further on‐site clinical services (Table 1). The majority, 89% (3130/3535) of the service users were TGW; 5% (192/3535) were transgender men and 6% (213/3535) were gender non‐conforming individuals. The mean age of our service users was 26 years with a standard deviation of 7.8. Fewer than half had completed high school (45%) and only 29% (1011/3535) were formally employed. Of those who reported earning an income (through employment, sex work, stipend or government social grants), 70% (1733/2487) earned less than R1000 (USD70) per month. Only 22% (761/3535) reported renting or owning their residence and 3.3% were homeless.

**Table 1:** Socio‐demographic characteristics of service‐users

	Frequency (*N* = 3535)	Percentage (%)
Gender		
Transgender woman	3130	88.5%
Transgender man	192	5.4%
Gender non‐conforming	213	6.0%
Sex assigned at birth		
Female	215	6.1%
Male	3320	93.9%
Age (years)		
<25	1714	48.5%
25–30	914	25.9%
31–35	466	13.2%
≥ 36	441	12.5%
Education		
No schooling	353	10.0%
Primary	34	1.0%
Grade 8–10	615	17.4%
Grade 11–12	1576	44.6%
Tertiary	913	25.8%
Unknown/refused	44	1.2%
Living situation		
Staying with family/friends	2497	70.6%
Rent or own house/apartment	761	21.5%
Streets/no place to live	115	3.3%
Unknown/refused	162	4.6%
Employed		
No	2362	66.8%
Yes	1011	28.6%
Unknown/refused	162	4.6%
Current monthly income *n* = 2487		
<R1000	1733	69.7%
R1000–R2999	253	10.2%
R3000–R4999	237	9.5%
R5000–R10,000	174	7.0%
>R10,000	90	3.6%

#### 3.1.2 Sexual and behavioural risk

Information on sexual and behavioural risk was collected for 78% (4417/5636) of the transgender individuals reached through outreach services (Table 2). Over a third (1517/4417) reported having condomless receptive anal sex in the last 12 months. Our service users reported low levels of violence, with 11% (483/4417) disclosing experiences of violence in the last 3 months. Most violence was experienced from current romantic partners, family members and neighbours. Close to a third (1226/4417) reported feeling sad, anxious and worried most of the time in the preceding 12 months. Most of our service users, 71% (3303/4664) had consumed more than four alcoholic drinks per day in the last month. When asked about their HIV risk perception, 59% (2622/4417) of the respondents reported to be at some risk/great risk of contracting HIV.

**Table 2:** Sexual and behavioural risk factors

	Frequency (*N* = 4417)	Percentage (%)
Condomless receptive anal sex in the last 12 months		
No	2829	64.0%
Yes	1517	34.3%
Unknown/refused	71	1.6%
Experience of sexual, psychological, physical violence		
No	3872	87.7%
Yes	483	10.9%
Unknown/refused	62	1.4%
Violence perpetrators *n =* 528^a^		
Current romantic partner	121	22.9%
Ex‐romantic partner	25	4.7%
Sex partner	63	11.9%
Sex work service user	41	7.8%
Family	97	18.4%
Employer/co‐worker	18	3.4%
Fellow transgender community member	16	3.0%
Neighbours	98	18.6%
Police/law enforcement	11	2.1%
Other	38	7.2%
Sad, depressed, anxious, worried much of the time in the last 12 months		
No	3107	70.3%
Yes	1226	27.8%
Unknown/refused	84	1.9%
Substance use in the last 30 days^a^ *n* = 4664		
Alcohol use (>4 drinks in a day)	3303	70.8%
Dagga (cannabis)	1219	26.1%
Nyaope (street drug blending heroin and other agents)	18	0.4%
Tik (methamphetamine)	85	1.8%
Heroin	39	0.8%
HIV risk perception		
No risk	487	11.0%
Some risk	1771	40.1%
Great risk	851	19.3%
Don't know	804	18.2%
N/A (known HIV‐positive status)	436	9.9%
Refused	68	1.5%

^a^Multiple‐response question.

#### 3.1.3 HIV status and utilization of gender‐affirming healthcare at enrolment

Before accessing services through our programme, 69% (2437/3535) of our clients did not know their HIV status. Of the service users who were previously diagnosed with HIV, 73% (241/329) were taking ART (Table 3). Among the respondents who had recently tested negative in the last 12 weeks, 8% (57/706) were taking PrEP. Of note, only 190 (5%) of service users reported any prior utilization of gender‐affirming healthcare services through the formal healthcare sector, with HT being the most utilized service.

**Table 3:** HIV status and utilization of gender‐affirming healthcare services at enrolment

	Frequency (*N* = 3535)	Percentage (%)
HIV status		
Unknown	2437	68.9%
Known negative (tested last 12 weeks)	706	20.0%
Known positive	329	9.3%
Refused	63	1.8%
HIV positive *n* = 329		
On ART	241	73.3%
If HIV negative *n* = 706		
On PrEP	57	8.1%
Prior utilization of gender‐affirming healthcare services		
No	3168	89.6%
Yes	161	4.6%
Unknown/refused	206	5.8%
Type of gender‐affirming healthcare received *n =* 190^a^		
Counselling/therapy	49	25.8%
Puberty blockers	8	4.2%
HT	65	34.2%
Surgery	2	1.1%
Birth control	44	23.2%
Other	22	11.6%

^a^Multiple‐response question as some transgender people utilized more than one service.

Abbreviations: ART, antiretroviral therapy; HT, hormone therapy; PrEP, pre‐exposure prophylaxis.

#### 3.1.4 HIV prevention cascade

**Figure 2:**
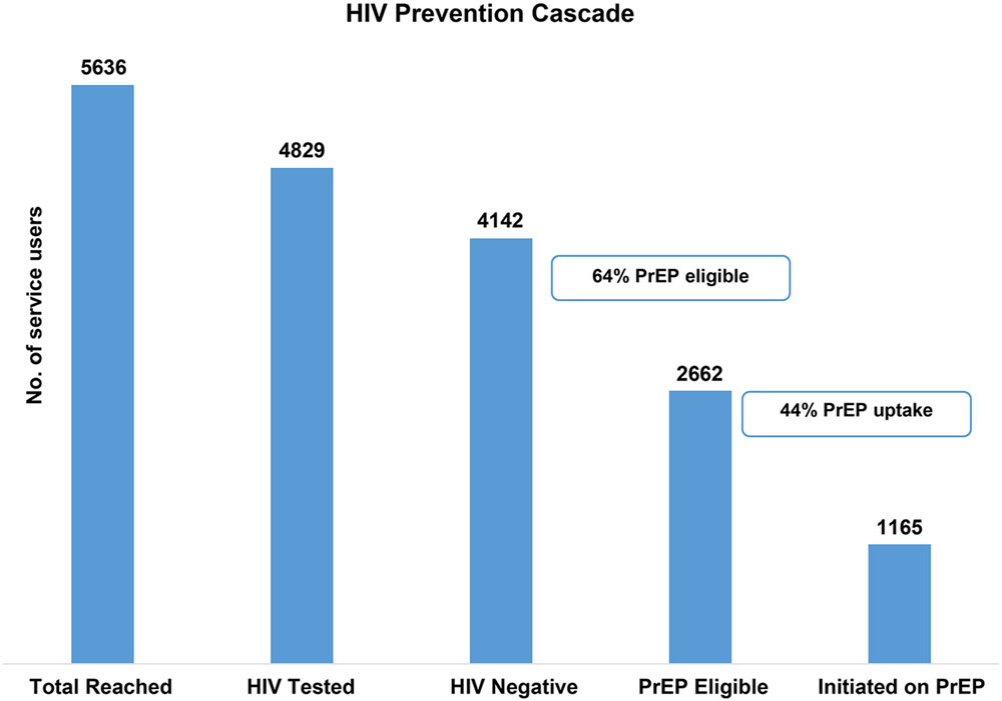
The HIV prevention cascade. Total number of people tested for HIV who received PrEP. Abbreviation: PrEP, pre‐exposure prophylaxis.

The HIV prevention cascade showed that 86% (4829/5636) of transgender people who were reached by peer educators through outreach accepted an HIV test (Figure 2). Of the 4142 (86%) who tested negative, 28% (1165/4142) accepted PrEP, this is 44% (1165/2662) of those who had indicated either some risk or great risk perception of contracting HIV. Most of the PrEP initiations occurred in the community, that is at the mobile clinic or gazebo (86%; 1001/1165).

There was no difference in the continued use of PrEP for 10 months or more between PrEP patients who were also taking HT and those not taking HT (OR = 1.04, *p* = 0.885) (Table 4). There was no evidence detected of association between age (*p* = 0.627) or gender identity (*p* = 0.753) and continued use of PrEP for 10 months or more, therefore, these variables were not included in a multivariate model.

**Table 4:** Hormone therapy and continued use of PrEP

	Total		Continued use of PrEP for 10 months or more		Discontinued PrEP before 10 months			
	(*N* = 287)		(*n* = 95)		(*n* = 192)			
	*n*	(%)	*n*	(%)	*n*	(%)	Crude OR (95% CI)	*p*‐value
Hormone therapy	80	27.87	27	33.75	53	66.3	1.04 (0.60–1.80)	0.885
No hormone therapy	207	72.13	68	32.85	139	67.2	1	

Abbreviations: CI, confidence interval; OR, odds ratio; PrEP, pre‐exposure prophylaxis.

### 3.2 HIV treatment cascade

**Figure 3:**
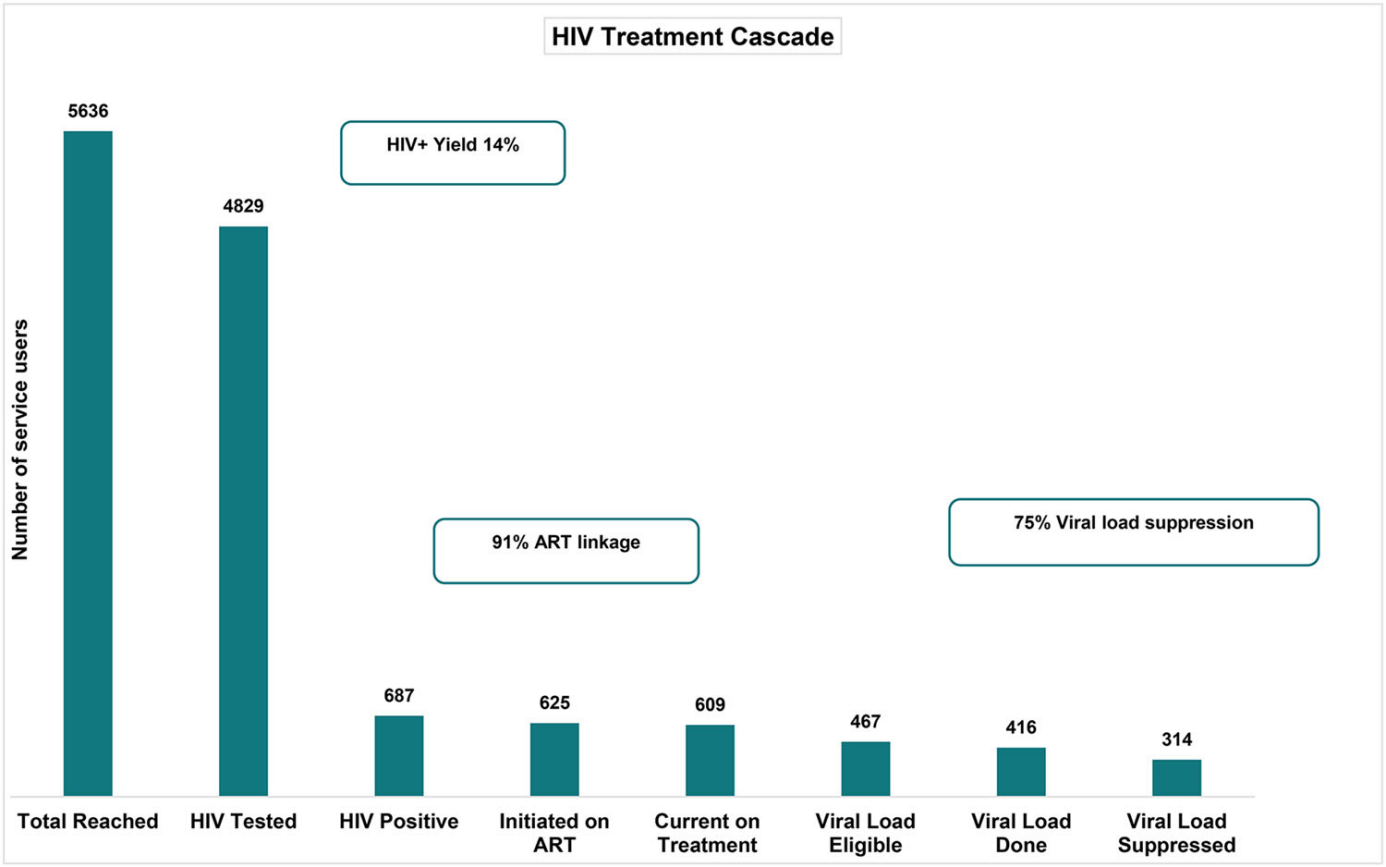
The HIV treatment cascade. Total number of people tested for HIV who received ART. Abbreviation: ART, antiretroviral therapy.

Among service users who tested for HIV, 14% (687/4829) were HIV positive, and 91% of those (625/687) received ART (Figure 3). Our programme retained 609 HIV‐positive people in care (97% of 625 initiated on ART), and 77% (467/609) of those were on ART for 6 months or more at the time of review, and therefore, eligible for a viral load test. The nurses collected blood samples for viral load testing from 89% (416/467) of those who were eligible for a viral load. The viral load suppression rate among service users who had a viral load test done was 75% (314/416) (HIV RNA <50 copies/ml).

### 3.3 HT and HIV services

**Figure 4:**
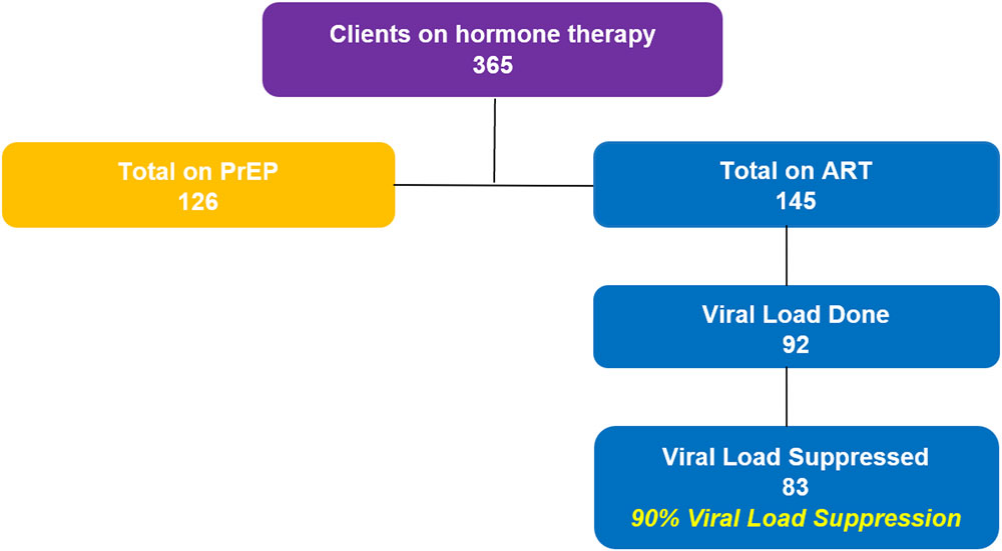
HIV services for transgender individuals on hormone therapy. Abbreviations: ART, antiretroviral therapy; PrEP, pre‐exposure prophylaxis.

We initiated 10% (365/3535) of service users on HT (Figure 4). HT provision was limited by the supply of pharmacy stock, and more than 200 people were on a waiting list at the time of review. Of those on HT, 35% (126/365) were linked to PrEP and 40% (145/365) were linked to ART. Viral load suppression among those on ART and taking HT was 90% (83/92), higher than the viral load recorded with the overall cohort.

ART clients who were receiving HT were three times more likely to have suppressed viral load (OR = 3.01, *p*<0.001) compared to those who were not receiving HT (Table 5). There was no difference detected in coverage of viral load test done between those receiving HT and those not receiving HT (OR = 1.76, *p* = 0.306). There was no detected evidence of an association between age (*p* = 0.407) or gender identity (*p* = 0.445) and viral load suppression, therefore, these variables were not included in the multivariate model. Geographic location was excluded from the model due to collinearity with the variable HT.

**Table 5:** Hormone therapy and viral load suppression

	Total		Viral load suppressed (≤50 copies/ml)		Viral load unsuppressed (>50 copies/ml)			
	(*N* = 472)		(*n* = 303)		(*n* = 169)			
	*n*	(%)	*n*	(%)	*n*	(%)	Crude OR (95% CI)	*p*‐value
Hormone therapy	98	20.76	80	81.63	18	18.4	3.01 (1.73–5.22)	<0.001
No hormone therapy	374	79.24	223	59.63	151	40.4	1	

Abbreviation: OR, odds ratio.

## 4 DISCUSSION

In this analysis of data from four transgender healthcare centres across South Africa, 88% of service users identified as TGW, higher than what has been reported in the South African transgender population size estimates, where 48% of transgender people identified as TGW [12]. The remainder of the participants identified as transgender men or gender non‐conforming. The high number of TGW in our programme compared to transgender men and gender non‐conforming individuals in our study is likely due to our programme's focus on TGW, identified as the priority population by the funder, PEPFAR through USAID. The low levels of high school completion (45% reported completing high school) echo previous studies which have shown that transgender people are often excluded from the education system [29, 30]. Participants reported a monthly income of less than R1000 (USD70), which is considerably below the minimum wage in South Africa of R20/hour [31] (approximately R3600 per month), and only one in five participants had their own place to live. These low levels are consistent with global findings regarding the barriers transgender people experience in accessing health‐determining resources, such as education, employment and housing, in addition to the stigma and discrimination they experience when accessing healthcare [29].

In our study, 36% of the service users reported condomless receptive anal sex in the last 12 months, consistent with what has been reported among transgender people in other countries [32]. A systematic review estimating per‐act HIV transmission reported that the risk of HIV transmission through condomless receptive anal sex was 138 infections per 10,000 exposures [33]. Full viral suppression with ART, known as U = U (Undetectable = Untransmittable), means that the risk of HIV transmission is very low, even without condom use [33]. Securing access to ART and promoting condom use is key. Fewer service users in our study reported experiences of violence compared to prior research in South Africa [34, 35] potentially due to social desirability bias in the data collection process. The high prevalence of violence experienced by transgender people has been well documented and UNAIDS has set targets to address the deeply entrenched stigma and discrimination which perpetuate violence against transgender people [32].

In South Africa, over 90% of the population know their HIV status and 75% of these are on HIV treatment [8]. Contrary to the general population, only a third of transgender individuals knew their HIV status in our study, of those who knew they were HIV positive, 73% were on HIV treatment, at the time they began accessing our services. These data are important in understanding the effectiveness of the HIV response for transgender people. It supports the need for innovative strategies, such as differentiated service delivery, to reach individuals at the highest risk of HIV infection [36].

Despite the progressive constitution in South Africa which prohibits discrimination based on sex, gender or sexual orientation [37], transgender people continue to face numerous social and structural barriers that hinder their access to quality healthcare services [38, 39]. Our study showed that one in 20 individuals had utilized gender‐affirming healthcare services. This low utilization could be a result of being denied healthcare, fear of stigma and discrimination from healthcare providers, which has been documented in previous studies and reports [5, 35, 40]. Among people who had received gender‐affirming services before utilizing our services, HT was the most sought‐after service. In the absence of gender‐affirming HT, it is common for TGW to use off‐label contraceptives, herbal medicines and other pills/substances without medical supervision [41]. The prevalence of unsupervised hormone use among transgender people ranged between 29% and 63% in New York City and Ontario, and was associated with health risks, such as hypercoagulability and decreased insulin sensitivity [41, 42]. Although there may be a considerable geographic variation of unsupervised hormone use, providing gender‐affirming healthcare in routine HIV programming could reduce these health risks among transgender people in South Africa.

Our study showed that PrEP was generally acceptable among service users who perceived themselves at risk of contracting HIV. The differentiated gender‐affirming healthcare services offered by our programme created trusting relationships between the clinical team and service users which may have enabled PrEP uptake, as was shown in San Francisco where gender‐affirming providers were described as facilitators for PrEP acceptability [17]. Most research has focused on the role of HT on PrEP uptake, without long‐term follow‐up, our study showed no significant association between HT and PrEP continued use for 10 months or more. Unlike ART, clients can cycle on and off PrEP. Barriers to the continued use of PrEP have been documented in several literatures [43, 44, 45]. Our clients reported the following barriers to continuation: pill fatigue, the stigma associated with HIV infection when PrEP is misidentified as ART and fear of disclosing PrEP use to partners. As HT becomes more readily available, there is a need to implement innovations to support the continued use of PrEP among transgender people.

Overall, 14% of all transgender individuals who received an HIV test tested positive, this HIV positive yield among our service users is lower than the HIV prevalence reported in previous studies [9]. The relatively low HIV‐positive yield in our study may be due to people who already knew their HIV status and declined testing without disclosing their status, or our programme reached relatively lower‐risk transgender people. Using the UNAIDS goal to reach 95‐95‐95 by 2030 as a benchmark, our programme achieved an 86‐91‐75 treatment cascade, linking a greater proportion of service users to ART and PrEP compared to the general population in South Africa [8]. These HIV prevention and treatment data are important in understanding the HIV epidemic among transgender people in South Africa, where data are still very sparse.

Our study findings are consistent with previous research, which suggests that gender‐affirming HT may improve engagement in the HIV care continuum among TGW [21, 46]. We found that service users who were on HT were three times more likely to be virally suppressed compared to those who did not have access to HT. Studies have reported improved quality of life and social functioning when HT [47, 48], which may enable individuals on ART to adhere to treatment with minimal interruption of treatment.

Our study is limited by the use of routine programme data, which is potentially of lower quality and completeness than data collected in a research project. To improve data reliability and validity, the programme provides in‐depth training to peer educators and clinical staff, many of whom are highly experienced in programme implementation. The data on demographics, sexual and behavioural risks were based on self‐report and may be skewed by recall or social desirability bias, poor understanding of the questions or reluctance to divulge sensitive personal information. One example of this is that contrary to expectations and reports from other studies, very few transgender people reported experiencing violence during the reporting period. As our programme matures, continuous staff training to improve screening and referrals for interpersonal violence is indicated. Incomplete data due to omission or respondents’ refusal to answer may have introduced bias. However, missing data were infrequent (<5% of records) and are unlikely to affect the interpretation of findings. Lastly, while the findings presented in this paper reflect the largest group of transgender service users in the country, they are not generalizable to different service delivery models in the country or region.

## 5 CONCLUSIONS

Our study addresses knowledge gaps in evidence‐based programming for transgender people in South Africa by providing data on a differentiated HIV and gender‐affirming healthcare service delivery model. We have shown that transgender people have low utilization of gender‐affirming healthcare in the public sector, leaving a high unmet demand for differentiated care. Overall, our findings indicate that differentiated gender‐affirming healthcare and HIV service delivery at a primary healthcare level is feasible in South Africa. The service user numbers reached and the high referral rate from outreach services into the clinics suggest high acceptability and a high potential for this programme model to engage the transgender population in HIV services. We have shown that providing gender‐affirming healthcare services, including HT, as a primary healthcare intervention significantly increases viral load suppression. Future reports from these transgender centres will provide critical information on the impact of differentiated services on retention in care and clinical outcomes. Studies to elucidate how the HIV treatment and prevention cascades differ for transgender people on HT at each point of the cascade are needed. Additional studies exploring the costing implications of integrating differentiated services into public sector primary healthcare would be beneficial.

## COMPETING INTERESTS

LLvdM: Community Global Advisory Committee for the Gilead Len4PrEP Study.

## AUTHORS’ CONTRIBUTIONS

RB conceptualized the study and drafted the initial version of the manuscript. CO, JN, VS, JS and LM contributed to data collection tool design, data collection and analysis. CO, MC and NH contributed to scientific aspects of the review, interpretation of results and revision of the paper. JJL and LLvdM provided editorial input to the manuscript. All authors reviewed and approved the manuscript for submission.

## FUNDING

The Wits RHI key populations programme is funded through the support of the American People through the U.S. President's Emergency Plan for AIDS Relief (PEPFAR) as implemented by the United States Agency for International Development 72067418CA00027 (USAID)/Southern Africa.

## DISCLAIMER

The contents of this review do not necessarily reflect the views of USAID or the United States Government.

## Data Availability

The data that support the findings of this study are available from the corresponding author upon reasonable request.
